# Antiradical Activity of Porphyrins with a Diisobornylphenol Fragment at the Macrocycle Periphery

**DOI:** 10.3390/molecules23071718

**Published:** 2018-07-14

**Authors:** Dmitrii V. Belykh, Lidiya I. Mazaletskaya, Nataliya I. Sheludchenko, Tatyana K. Rocheva, Irina S. Khudyaeva, Evgeny V. Buravlev, Olga V. Shchukina, Irina Yu. Chukicheva

**Affiliations:** 1 Institute of Chemistry, Komi Scientific Centre, Ural Branch of the Russian Academy of Sciences, 48 ul. Pervomayskaya, 167000 Syktyvkar, Russia; belykh-dv@chemi.komisc.ru (D.V.B.); khudyaeva_is@mail.ru (I.S.K.); eugeneburavlev@gmail.com (E.V.B.); sukrusheva@mail.ru (O.V.S.); chukichevaiy@mail.ru (I.Y.C.); 2 Emanuel Institute of Biochemical Physics, Russian Academy of Sciences, 4 ul. Kosygina, 119334 Moscow, Russia; mazaletskaya2011@yandex.ru (L.I.M.); nish48@mail.ru (N.I.S.)

**Keywords:** tetra(*meso*-aryl)porphyrins, chlorophyll derivatives, 2,6-diisobornylphenol, antiradical activity, stoichiometric inhibition coefficient, inhibited ethylbenzene oxidation

## Abstract

This article focuses on the antiradical activity of a number of 2,6-diisobornylphenol-porphyrin conjugates with various spacers between the porphyrin and phenolic fragments in the model reaction of ethylbenzene oxidation initiated by azoisobutyric acid dinitrile. The study has shown that the electronic effects of the groups directly related to the 2,6-diisobornylphenol fragment exert the predominant influence both on the reactivity of the phenolic hydroxyl group in interaction with free radicals and on the antiradical activity of the molecule as a whole. The antiradical activity of the molecule is generally less affected by the nature of the substituents in the porphyrin macrocycle, mainly due to a change in the stoichiometric inhibition coefficient in the presence of relatively easily oxidizable groups. It was found that the length of the spacer between the porphyrin and phenolic fragments does not affect the antiradical activity of the conjugate.

## 1. Introduction

**Figure 1:**
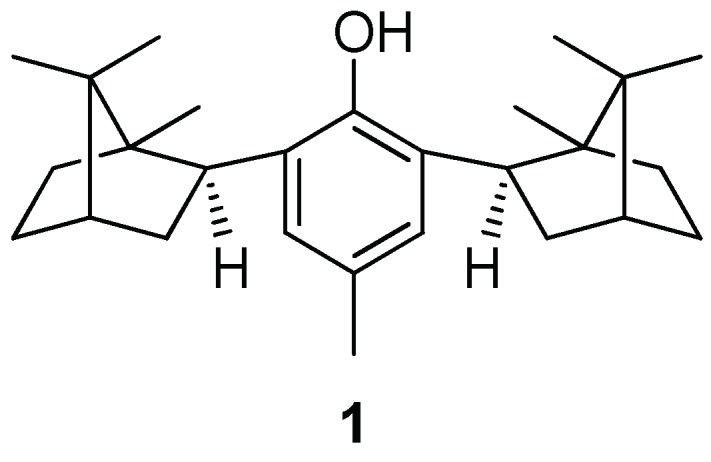
Structure of 2,6-diisobornyl-4-methylphenol **1**.

Hybrid antioxidants (AO) with radical scavenging activity (RSA) are known as promising compounds for the development of new biologically active substances. Such hybrid molecules can combine several reaction centers capable of inhibiting oxidative processes by various mechanisms and exhibiting an intramolecular synergistic effect [1,2,3]. In addition, examples of molecules combining an antioxidant reaction center with a fragment providing targeted delivery in the body or the ability to structure interactions with the biosystem are described [1]. 2,6-Diisobornyl-4-methylphenol **1** (Figure 1) is a compound with a wide spectrum of biological activity, while the effects it exhibits in most cases are associated with its antioxidant properties [4,5,6]. On the other hand, it is known that the lipophilic free bases of porphyrins and their complexes with metals can be accumulated in the lipid bilayer of cell membranes [7,8] and are capable of inactivating free radicals [9,10,11], so the study of anti-radical properties of porphyrins remains relevant [12]. The insertion of fragments of molecules having their own antioxidant activity (AOA) to the periphery of the porphyrin macrocycle can have a significant effect on the AOA molecule as a whole. We have previously shown that symmetrically substituted tetra(*meso*-aryl)porphyrins as well as derivatives of chlorophyll *a* with 2,6-diisobornylphenol fragments on the periphery of the macrocycle exhibit AOA and RSA and these properties are in all cases due to the presence of the phenol fragment in the molecule for all types of porphyrin [9,13,14,15,16]. In this connection, the information about the porphyrin macrocycle effect on the phenolic hydroxyl groups reactivity in the interaction with free radicals is of a great interest for the design of such hybrid AO molecules. In this paper, we investigate the RSA of natural and synthetic porphyrins containing 2,6-diisobornylphenol fragment differing in the structure of the macrocycle as well as the location and mode of conjugation.

## 2. Results and Discussion

**Scheme 1:**
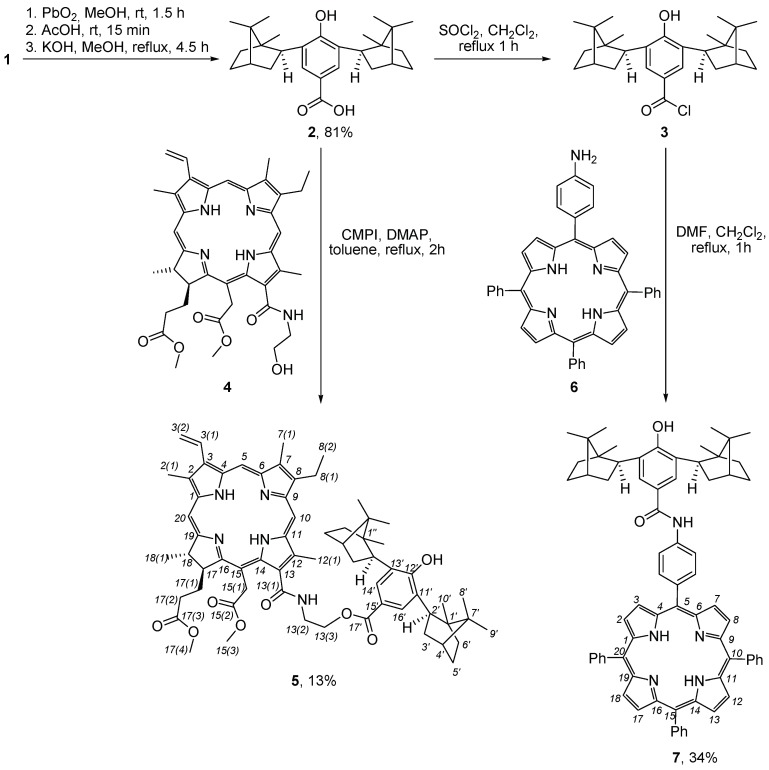
Synthesis of porphyrin conjugates with 2,6-diisobornylphenol.

The synthesis of the target compounds is shown in Scheme 1. Starting from *p*-cresol **1,** in accordance with the method from Ref. [17], acid **2** was synthesized, from which the acid chloride **3** was prepared in situ. The conjugate **5** was synthesized by porphyrin **4** hydroxyl group acylation using acid **2** activated with 2-chloro-*N*-methylpyridinium iodide (CMPI). The reaction of porphyrin **6** with acid chloride **3** gave the conjugate **7**.

The structure of the first synthesized compounds **5** and **7** was confirmed by NMR, IR, and UV-Vis spectroscopy as well as by mass spectrometry.

**Figure 2:**
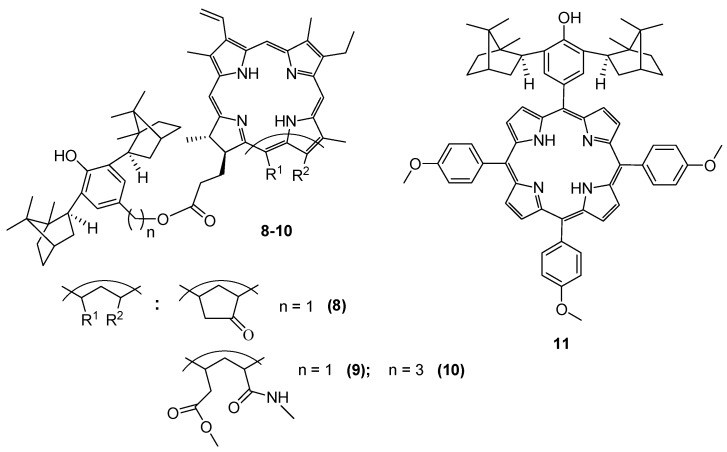
Structure of the conjugates **8**–**11**.

Conjugates **5**, **7,** and previously synthesized [15,18] derivatives **8**–**11** (Figure 2) were used for the comparative study of RSA.

**Figure 3:**
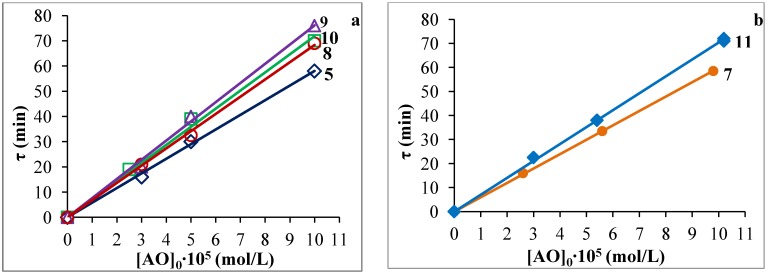
Dependence of induction period of inhibited ethylbenzene oxidation on the initial concentration of compounds **5**, **8**–**10** (**a**) and **7**, **11** (**b**). Temperature 333 K, *W*_i_ = 5 × 10^–8^ mol/L·s.

**Figure 4:**
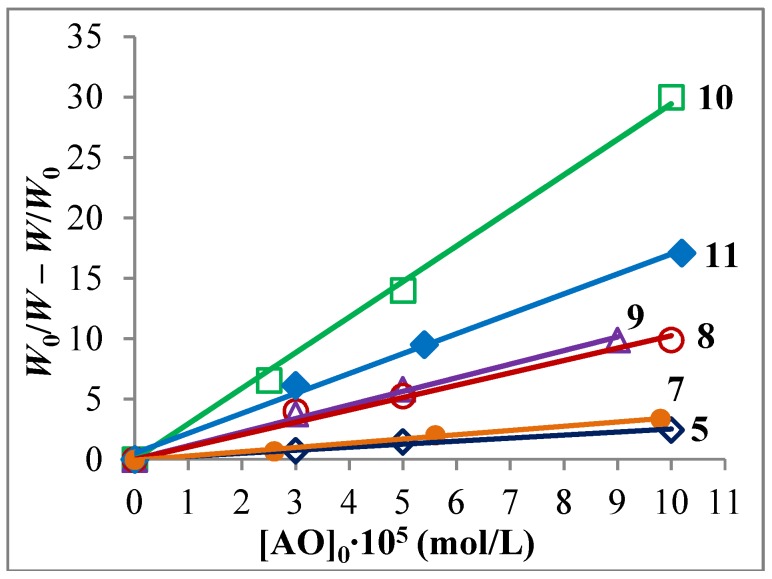
Dependence of the initial rate of inhibited ethylbenzene oxidation on the initial concentration of compounds **5**, **7**–**11** in the coordinates of the equation *W*_0_/*W* − *W*/*W*_0_ = *fk*_inh_[AO]_0_/*k*_r_^0.5^*W*_i_^0.5^. Temperature 333 K, *W*_i_ = 5 × 10^−8^ mol/L·s.

The inhibitory effect of porphyrins with phenolic substituents was studied in the model reaction of ethylbenzene oxidation initiated by dinitrile of azoisobutyric acid using the volumetric method of oxygen uptake [19]. Ethylbenzene with dissolved AO was pre-thermostated and then initiator was added. The induction period (τ) and the initial oxygen absorption rate in the presence of AO (W) were determined from the kinetic curves. It is clear from Figure 3 that the induction period depends linearly on the initial concentration of AO. The stoichiometric inhibition coefficient was calculated from the slope of the straight lines tangent (Figure 3) using the equation:

*f* = τ*W*_i_/[AO]_0_, (1)

where *W*_i_ is the initiation rate, [AO]_0_ is the AO concentration. To calculate the rate constants of the interaction of AO with peroxyl radicals (*k*_inh_), the values of the initial oxidation rates were represented (Figure 4) in the coordinates of the equation [20]:

*W*_0_*/W* − *W/W_0_* = *fk*_inh_[AO]_0_/*k*_r_^0.5^*W*_i_^0.5^, (2)

where W_0_ and *W* are the oxidation rates in the absence and in the presence of AO respectively, *k*_r_ is the rate constant of the quadratic recombination of peroxy radicals (*k*_r_ = 1.9 × 10^7^ L/mol*∙*s) [21]. The value of *fk*_inh_ was determined from the slope of the straight lines presented in Figure 4. Table 1 presents the calculated values of the stoichiometric coefficient *f* and the inhibition parameter *fk*_inh_, as well as the *k*_inh_ values. 2,6-Diisobornyl-4-methylphenol **1** (Figure 1) was used as the reference compound.

The *k*_inh_ value for compound **10** with 2,6-diisobornylphenol fragment maximally removed from the porphyrin macrocycle is close to *k*_inh_ value for phenol **1**. This fact demonstrates the absence of the macrocycle influence on the reactivity of phenolic hydroxyl group. The *k*_inh_ values obtained for compounds **5**, **7**, **9**, and **11** indicate the prevailing influence of groups directly related to the 2,6-diisobornylphenol fragment on the reactivity of its hydroxyl group in the reaction with free radicals. The smallest *k*_inh_ values were observed for compounds **5** and **7** containing electron-withdrawing ester (compound **5**) and amide (compound **7**) groups, the *k*_inh_ values of these compounds being close, despite significant differences in the structure of the porphyrin moiety. Compounds **8**, **9** with similar spacer structure and notable differences of porphyrin parts, have close *k*_inh_ values too. Both of the porphyrins have an electron-withdrawing acyl group separated from the phenol fragment by one methylene group, so *k*_inh_ is significantly larger than that of compounds **5** and **7**, but significantly smaller than in compound **1** that does not contain a porphyrin macrocycle in the molecule, and conjugate **10** in which the acyl group is separated from the phenolic aromatic ring by three methylene units. In spite of the close proximity of the terpenylphenol fragment to the porphyrin macrocycle, it is interesting that the *k*_inh_ of compound **11** is close to that of compound **1**. It is known that the methyl group of compound **1** has a weak *+M*-effect due to hyperconjugation and +*I*-effect due to the greater electronegativity of the benzene ring *sp*^2^-hybrid carbon atom compared to the *sp*^3^-hybrid carbon atom of the methyl group. Regarding the porphyrin macrocycle, it has an −*I*-effect, and should reduce the reactivity of the hydroxyl group of the phenolic moiety. However, the electron-donor properties of the porphyrin macrocycle *meso*-aryl substituents methoxy groups seem to significantly reduce the −*I*-effect. A similar effect was observed for porphyrins with unsubstituted phenol as a substituent that determines RSA [22]. Thus, the order of *k*_inh_ increase: **5** ≈ **7** < **9** ≈ **8** < **11** ≈ **10** ≈ **1** indicates the predominant influence of groups related directly to the 2,6-diisobornylphenol fragment on the reactivity of phenolic hydroxyl in interaction with free radicals.

Similar patterns were observed for other porphyrin conjugates with terpenylphenols: the main inhibitory effect of porphyrin molecules with phenolic substituents is due precisely to the hydroxyl group of the phenol fragment and, consequently, their RSA essentially depends on the composition and location of the substituents in the phenol fragment [22,23,24,25]. The structure of the porphyrin part of the conjugate has a significant effect on the value of the stoichiometric inhibition coefficient (*f*). The value of *f* for most of the conjugates studied is higher than for the reference compound (except for **5** and **7**). As compared to **1**, the increasing *f* for compounds **8**–**11**, is apparently due to the presence of relatively easily oxidizable groups in the porphyrin macrocycle. It is most likely the amide group at position 13(2) in compounds **9** and **10**, methylene group at position 13(2) in the conjugate with pyrophoeophorbide *a* fragment **8**, and *p*-methoxyphenyl substituents in unsymmetrically substituted tetra(*meso*-aryl)porphyrin **11**.

The effect of structural factors on the total RSA of terpenylphenol-chlorin conjugates, reflecting the inhibition parameter *fk*_inh_, is generally analogous to the effect of structural factors on the reactivity of the hydroxyl group of the phenolic fragment. A very high RSA is observed in the case where the phenolic substituent is directly connected to the porphyrin macrocycle (compound **11**) via the *p*-position: the value of the *fk*_inh_ parameter is close to the analogous parameter of 2,6-diisobornyl-4-methylphenol **1** (Table 1). The presence of an electron-withdrawing amide group in the *p*-position (compound **7**) leads to a sharp decrease in the *fk*_inh_ parameter, which is ≈6.3 times lower in comparison with **1** and is ≈5.2 times lower than that of porphyrin **11**. Similar results were obtained for conjugates of chlorophyll *a* derivatives **5**, **8**–**10**. As can be seen from the data in Figure 4 and Table 1, this conjugates are very markedly different in terms of RSA which varies depending on the spacer that connects the phenolic moiety to the *p*-position with the chlorin ring. In spite of the fact that in all the considered cases, the spacer includes a carbonyl group, the degree of the remoteness of this group from the benzene ring affects the activity of the hydroxyl group significantly. For example, with the maximum removal of the carbonyl group from the phenyl fragment (compound **10**) the *fk*_inh_ parameter is even higher than for **1**. In the case of conjugates **8** and **9** in which the phenol fragment is separated from the carbonyl by the -CH_2_O- group, a certain decrease in the *fk*_inh_ parameter is observed, and its value is practically independent of substituents in the chlorin ring (Table 1). Finally, in the case of carbonyl group bonded directly to the benzene ring carbon atom located in the *p*-position regarding the hydroxyl group (compound **5**), the *fk*_inh_ parameter is *fk*_inh_ = 2.4 × 10^4^ L/mol·s only, more than an order of magnitude different from the most effective free radical oxidation inhibitor **10** and much lower than the *fk*_inh_ parameter of the reference compound **1**.

## 3. Materials and Methods

### 3.1. Investigation of the Compounds Obtained

IR spectra were recorded on a Shimadzu IR Prestige 21 FTIR spectrometer in KBr tablets. Electronic spectra were recorded on a Shimadzu UV-1700 spectrometer in quartz cuvettes 10 mm thick (reference: dichloromethane). The ^1^H and ^13^C NMR spectra were recorded on a Bruker Avance II spectrometer (operating frequency 300 and 75 MHz) for solutions of substances in CDCl_3_. ESI mass spectra were recorded on a Thermo Finnigan LCQ Fleet. The reactions progress was monitored by thin layer chromatography on Sorbfil plates. Aluminum oxide 40/200 μm of “h” grade was used for column chromatography.

### 3.2. Chemicals and Reagents

The following solvents were used in the research: solvents of the following manufacturers: toluene *Component reaktiv* (Moscow, Russia), chloroform, carbon tetrachloride *Ecos* (Moscow, Russia), methanol *Vekton* (Saint-Petersburg, Russia). Dimethylformamide (DMF) *Reachem* (Moscow, Russia) was used without any additional purification, purification of dichloromethane *Ecos* (Moscow, Russia) was carried out according to the known method [26]. 2-Chloro-*N*-methylpyridinium iodide (CMPI) *Acros* (NJ, USA)*,* 4-dimethylaminopyridine (DMAP) *Vekton* (Saint-Petersburg, Russia), thionyl chloride *Reactiv* (Saint-Petersburg, Russia), anhydrous sodium sulfate *Chemreactivsnab* (Ufa, Russia), hydrochloric acid *Sigma Tek* (Khimki, Russia) were used.

### 3.3. Synthesis of Conjugates and Their Precursors

4-Methyl-2-{(1*R*,2*S*,4*S*)-1,7,7-trimethylbicyclo[2.2.1]heptan-2-yl}-6-{(1*S*,2*R*,4*R*)-1,7,7-trimethylbicyclo[2.2.1]heptan-2-yl}phenol (**1**) was obtained according to [27].

4-Hydroxy-3-{(1*R*,2*S*,4*S*)-1,7,7-trimethylbicyclo[2.2.1]heptan-2-yl}-5-{(1*S*,2*R*,4*R*)-1,7,7-trimethylbicyclo[2.2.1]heptan-2-yl}benzoic acid (**2**) was obtained according to [17].

Methyl-3-{(7*S*,8*S*)-18-ethyl-3-((2-hydroxyethyl)carbamoyl)-5-(2-methoxy-2-oxoethyl)-2,8,12,17-tetramethyl-13-vinyl-7*H*,8*H*-porphyrin-7-yl}propanoate (**4**) was obtained according to [28].

2-{(7*S*,8*S*)-18-Ethyl-5-(2-methoxy-2-oxoethyl)-7-(3-methoxy-3-oxopropyl)-2,8,12,17-tetramethyl-13-vinyl-7*H*,8*H*-porphyrin-3-carboxamido}ethyl 4-hydroxy-3-{(1*R*,2*S*,4*S*)-1,7,7-trimethylbicyclo[2.2.1]heptan-2-yl}-5-{(1*S*,2*R,*4*R*)-1,7,7-trimethylbicyclo[2.2.1]heptan-2-yl}benzoate (**5**). A solution of porphyrin **4** (42 mg, 0.06 mmol), CMPI (32 mg, 0.13 mmol), acid **2** (32 mg, 0.08 mmol) and DMAP (38 mg, 0.31 mmol) in 15 mL of toluene was boiled for 1 h under reflux. Then CMPI (32 mg, 0.13 mmol), acid **2** (31 mg, 0.08 mmol) was added and the mixture was boiled for 1 h. After cooling the reaction mixture was diluted with dichloromethane (100 mL) and washed with a 5% HCl solution to remove excess of DMAP and CMPI conversion products and then with distilled water until neutral washing water, dried with anhydrous Na_2_SO_4_, and evaporated under reduced pressure. The residue after evaporation was chromatographed on alumina (eluent: CH_2_Cl_2_-CH_3_OH, 60:0 → 30:1). The eluate containing the desired product was evaporated under reduced pressure. 8 mg (13%) of compound **5** were obtained as a dark blue-green crystalline powder. UV-Vis (CH_2_Cl_2_), *λ*_max_, nm(%): 663.0 (33), 607.0 (4), 558.5 (2), 528.0 (3), 501.0 (10), 401.5 (100). *m*/*z* (ESI): for [M]^+^ C_65_H_81_N_5_O_8_, calculated: 1060.7, found: 1060.6. ^1^H-NMR (300 MHz, CDCl_3_): *Signals of the terpenylphenol fragment:* 0.64, 0.65,0.76, 0.78 (all s, 18H, CH_3_-8′,8′′, CH_3_-9′,9′′, CH_3_-10′,10′′), 1.49–1.71, 1.23–1.43, 0.98–0.84 (all m, 12H, H-3′,3′′, H-4′,4′′, CH_2_-5′,5′′, CH_2_-6′,6′′), 1.84–2.19 (m, 1H, H-3′,3′′), 2.84–3.00 (m, 2H, H-2′,2′′), 5.32 (br s, 1H, ArOH), 7.95 (s, 2H, H-14′,16′). *Signals of the porphyrin fragment:* −1.73 (br s, 1H, I-NH), −1.53 (br s, 1H, III-NH), 1.72–1.82 (m, 6H, CH_3_-18(1), CH_3_-8(2)), 2.19–2.30 (m, 2H, CH_2_-17(2)), 2.43–2.84 (m, 2H, CH_2_-17(1)), 3.35 (s, 3H, CH_3_-2(1)), 3.53 (s, 3H, CH_3_-7(1)), 3.55 (s, 3H, CH_3_-12(1)), 3.65 (s, 3H, CH_3_-17(4)), 3.81 (s, 3H, CH_3_-15(3)), 3.79–3.93 (m, 2H, CH_2_-8(1)), 4.32–4.37 and 3.98–4.15 (m, by 2H, CH_2_-13(2)), 4.41 (br d, 1H, *J* = 9.2, H-17), 4.51 (q, 1H, *J* = 7.3, H-18), 4.70–4.87 (m, 2H, CH_2_-13(3)), 5.31 (d, 1H, *J* = 19.3, H-15(1)^A^), 5.60 (d, 1H, *J* = 19.3, H-15(1)^A^), 6.17 (d, 1H, *J* = 11.0, H*_cis_*-3(2)), 6.38 (d, 1H, *J* = 17.4, H*_trans_*-3(2)), 6.80–6.92 (m, 1H, NH-13(1)), 8.05–8.18 (m, 1H, H-3(1)), 8.84 (s, 1H, H-20), 9.66 (s, 1H, H-5), 9.71 (s, 1H, H-10). ^13^C-NMR (75 MHz, CDCl_3_): *Signals of the terpenylphenol fragment:* 12.58 (C(10′,10′′)), 21.01 (C(9′,9′′)), 21.15 (C(8′,8′′)), 27.41 (C(5′,5′′)), 34.01 (C(3′,3′′)), 39.98 (C(6′,6′′)), 45.22 (C(4′,4′′)), 46.01 (C(2′,2′′)), 48.19 (C(7′,7′′)), 50.00 (C(1′,1′′)), 120.59 (C(13′)), 127.70 (C(14′,16′)), 128.80, 129.51 (C(11′,15′)), 158.69 (C(12′)). *Signals* *of* *the* *porphyrin* *fragment:* 11.35 (C(7(1))), 11.94 (C(2(1))), 12.17 (C(12(1))), 17.78 (C(8(2))), 19.70 (C(8(1))), 23.06 (C(18(1))), 29.72 (C(17(2))), 31.17 (C(17(1))), 37.94 (C(15(1))), 40.12 (13-CONHCH_2_CH_2_), 49.28 (C(18)), 51.62 (C(17(4))), 52.18 (C(15(3))), 53.09 (C(17)), 93.62 (C(20)), 98.82 (C(5)), 101.47 (C(10)), 102.27 (C(15)), 121.63 (C(3(2))), 127.86 (C(13)), 129.69 (C(3(1))), 129.72 (C(12)), 130.22 (C(2)), 134.55 (C(7)), 134.85 (C(4)), 134.93 (C(3)), 136.08 (C(11)), 138.95 (C(1)), 144.76 (C(8)), 149.10 (C(14)), 154.33 (C(9)), 166.66 (C(6)), 167.22 (C(16)), 168.91 (C(19)), 169.71 (13-CON), 173.52 (C(17(3)), (13-CONHCH_2_CH_2_OCOAr)), 173.90 (C(15(2))). IR spectrum, (KBr), cm^–1^: 3314 (NH), 2953, 2876, 2727 (CH_2_, CH_3_), 1734 (C=O, ester), 1665 (C=O, «amide–I»), 1601 («chlorin band»), 1510 («amide–II»).

4-(10,15,20-Triphenylporphyrin-5-yl)aniline (**6**) was obtained according to [29]. 

4-Hydroxy-3-{(1*R*,2*S*,4*S*)-1,7,7-trimethylbicyclo[2.2.1]heptan-2-yl}-5-{(1*S*,2*R*,4*R*)-1,7,7-trimethylbicyclo[2.2.1]heptan-2-yl}-*N*-{4-(10,15,20-triphenylporphyrin-5-yl)phenyl}benzamide (**7**). Thionyl chloride (50 μL, 0.63 mmol) was added to a solution of acid **2** (65 mg, 0.16 mmol) in dichloromethane (10 mL) and refluxed for 1 h. The solvent and the volatile components were then removed under reduced pressure. Dichloromethane (10 mL) and DMF (0.1 mL) was added to the obtained in situ acid chloride **3**, then solution of porphyrin **6** (30 mg, 0.05 mmol) in dichloromethane (15 mL) was added. The reaction mixture was refluxed for 1 h, then cooled, a 5% HCl solution was added and organic phase washed with water until a neutral reaction of the wash water. The resulting solution was dried over anhydrous Na_2_SO_4_, and the solvent was removed under reduced pressure. The reaction products were separated by column chromatography on alumina (eluent: CCl_4_, CHCl_3_). 12 mg (34%) of compound **7** were obtained as a dark red-violet crystalline powder. UV-Vis (CH_2_Cl_2_), *λ*_max_, nm (%): 666.5 (1), 594.5 (1), 551.0 (1), 515.5 (3), 420.0 (100). *m/z* (ESI): for [MH]^+^ C_71_H_68_N_5_O_2_, calculated: 1022.5, found: 1022.9. ^1^H-NMR (300 MHz, CDCl_3_): *Signals of the terpenylphenol fragment:* 0.88 (s, 6H, CH_3_-10′,10′′), 0.94 (s, 6H, CH_3_-9′,9′′), 1.07 (s, 6H, CH_3_-8′,8′′), 1.30–2.01 (m,12H, H-3′,3′′,4′,4′′, CH_2_-5′,5′′, CH_2_-6′,6′′), 2.43–2.55 (m, 2H, H-3′,3′′), 3.14 (br m, 2H, *J* = 8.3, H-2′,2′′),5.34 (s, 1H, OH), 7.90 (s, 2H, H-14′,16′). *Signals* *of* *the* *porphyrin* *fragment:* −2.73 (br s, 2H, NH-pyrrole), 7.81 (br s, 9H, H-10(3), H-10(4),H-10(5),H-15(3),H-15(4),H-15(5),H-20(3),H-20(4),H-20(5)), 8.06 (d, 4H, *J* = 8.2, H-5(3),H-5(5)), 8.13 (br s, 1H, NH), 8.25–8.27 (m, 8H, H-5(2),H-5(6),H-10(2),H-10(6),H-15(2),H-15(6),H-20(2), H-20(6)), 8.89 (br s, 6H, H-2,8,12,13,17,18), 8.94 (d, 2H, *J* = 4.6, H-3,7). ^13^C-NMR (75 MHz, CDCl_3_): *Signals of the terpenylphenol fragment:* 12.76 (C(10′,10′′)), 20.48 (C(9′,9′′)), 21.44 (C(8′,8′′)), 27.58 (C(5′,5′′)), 34.29 (C(3′,3′′)), 40.18 (C(6′,6′′)), 45.48 (C(4′,4′′)), 46.30 (C(2′,2′′)), 48.49 (C(7′,7′′)), 50.26 (C(1′,1′′)), 124.94 (C(14′,16′)), 129.25 (C(11′,13′)), 157.64 (C(12′)), 138.02 (C(15′)). *Signals* *of* *the* *porphyrin* *fragment:* 118.42 (C(5(3))),(C(5(5))), 119.58, 120.17 (C(5,10,15,20)), 126.70 (C(10(3))),(C(10(5))), (C(15(3))),(C(15(5))),(C(20(3))),(C(20(5))), 127.73 (C(10(4))),(C(15(4))),(C(20(4))), 130.06–131.97 (C(1)),(C(2)),(C(3)),(C(4)),(C(60),(C(7)),(C(8)),(C(9)),(C(11)),(C(12)),(C(13)),(C(14)),(C(16)),(C(17)), (C(18)),(C(19))), 134.57, 135.18 (C(5(2))),(C(5(6))),(C(10(2))),(C(10(6))),(C(15(2))),(C(15(6))),(C(20(2))), (C(20(6))), 138.10 (C(5(4))), 142.21 (C(5(1))),(C(10(1))),(C(15(1))),(C(20(1))), 166.90 (–NHCO). IR spectrum, (KBr), cm^–1^: 3590 (OH), 3316 (NH), 2951, 2876, 2708 (CH_2_, CH_3_), 1641 (C=O, «amide–I»), 1597(«chlorin band»), 1516 («amide–II»).

(3*S*,4*S*)-{*O*-[(4-Hydroxy-3-{(1*S*,2*R*,4*R*)-1,7,7-trimethylbicyclo[2.2.1]heptan-2-yl}-5-{(1*R*,2*S*,4*S*)-1,7,7-trimethylbicyclo[2.2.1]heptan-2-yl}phenyl)methyl]}-4,8,13,18-tetramethyl-20-oxo-9-ethenyl-14-ethylphorbin-3-propanoate (**8**) was obtained according to [15].

Methyl-2-{*O*-[(4-hydroxy-3-{(1*S*,2*R*,4*R*)-1,7,7-trimethylbicyclo[2.2.1]heptan-2-yl}-5-{(1*R*,2*S*,4*S*)-1,7,7-trimethylbicyclo[2.2.1]heptan-2-yl}phenyl)methyl]carbamoylethyl}-18-[(*N*-methyl)carbamoyl] chlorin-20-yl)acetate (**9**) was obtained according to [15].

Methyl-2-(*O*-3-[(4-hydroxy-3-{(1*S*,2*R*,4*R*)-1,7,7-trimethylbicyclo[2.2.1]heptan-2-yl}-5-{(1*R*,2*S*,4*S*)-1,7,7-trimethylbicyclo[2.2.1]heptan-2-yl}phenyl)propyl]carbamoylethyl}-18-[(*N*-methyl) carbamoyl]chlorin-20-yl)acetate (**10**) was obtained according to [15].

2-{(1*R*,2*S*,4*S*)-1,7,7-Trimethylbicyclo[2.2.1]heptan-2-yl}-6-{(1*S*,2*R*,4*R*)-1,7,7-trimethylbicyclo[2.2.1]heptan-2-yl}-4-{10,15,20-tris(4-methoxyphenyl)porphyrin-5-yl}phenol (**11**) was obtained according to [18]. 

### 3.4. RSA Estimation of Porphyrins and Chlorins with the 2,6-diisobornylphenol Fragment at the Periphery of the Macrocycle

*RSA* was estimated in a model reaction of the ethylbenzene initiated oxidation at 333 K in an air atmosphere. Dinitrile of azoisobutyric acid was used as the initiator of free radicals, the initiation rate was *W*_i_ = 5 × 10^−8^ mol/L·s. Ethylbenzene with a dissolved antioxidant was pre-thermostated. After that, an initiator additive was added. The oxygen absorption kinetics was measured with a volumetric unit [19]. Statistical analysis was performed using the conventional method of variation statistics. The calculations were made using Microsoft Office Excel-2007 program. The measurement error did not exceed 6%. 

## 4. Conclusions

Thus, the estimation of the antiradical activity of the conjugates presented in the present study showed that the electronic effects of groups directly connected with the 2,6-diisobornylphenol moiety generally exert the predominant effect on both the reactivity of phenolic hydroxyl in interaction with free radicals and the antiradical activity of the molecule. The nature of the substituents in the porphyrin macrocycle affects the activity of the compounds to a much lesser extent, due mainly to a change in the stoichiometric inhibition coefficient in the presence of relatively easily oxidisable groups; the length of the spacer between the porphyrin and the terpenylphenol fragment has no effect on the antiradical activity of the conjugates.

## Figures and Tables

**Table 1:** The values of the stoichiometric coefficient *f*, the inhibition parameter *fk*_inh_, the rate constant of the reaction with the peroxy radicals ethylbenzene *k*_inh_ (temperature 333 K).

Compound	*f*	*fk*_inh_∙10^−4^ (L/mol·s)	*k*_inh_∙10^−4^ (L/mol·s)
**1**	1.8	20.7	11.5
**5**	1.7	2.4	1.4
**7**	1.8	3.3	1.8
**8**	2.0	10	5.0
**9**	2.3	11	4.8
**10**	2.2	29	13.2
11	2.1	17	8.1
